# Epigallocatechin-3-gallate Enhances Cognitive and Memory Performance and Protects Against Brain Injury in Methionine-induced Hyperhomocysteinemia Through Interdependent Molecular Pathways

**DOI:** 10.1007/s12640-022-00605-4

**Published:** 2022-11-17

**Authors:** Mostafa D. Mostafa, Magda A ElKomy, Azza I. Othman, Maggie E. Amer, Mohamed A. El-Missiry

**Affiliations:** Zoology Department, Faculty of Science, Mansoura University, Mansoura, Egypt

**Keywords:** Methionine, Epigallocatechin-3-gallate, Neurotoxicity, Morris water maze, Hyperhomocysteinemia

## Abstract

Brain injury and cognitive impairment are major health issues associated with neurodegenerative diseases in young and aged persons worldwide. Epigallocatechin-3-gallate (EGCG) was studied for its ability to protect against methionine (Met)-induced brain damage and cognitive dysfunction. Male mice were given Met-supplemented in drinking water to produce hyperhomocysteinemia (HHcy)-induced animals. EGCG was administered daily concurrently with Met by gavage. EGCG attenuated the rise in homocysteine levels in the plasma and the formation of amyloid-β and tau protein in the brain. Cognitive and memory impairment in HHcy-induced mice were significantly improved by EGCG administration. These results were associated with improvement in glutamate and gamma-aminobutyric acid levels in the brain. EGCG maintained the levels of glutathione and the activity of antioxidant enzymes in the brain. As a result of the reduction of oxidative stress, EGCG protected against DNA damage in Met-treated mice. Moreover, maintaining the redox balance significantly ameliorated neuroinflammation evidenced by the normalization of IL-1β, IL-6, tumor necrosis factor α, C-reactive protein, and IL-13 in the same animals. The decreases in both oxidative stress and inflammatory cytokines were significantly associated with upregulation of the antiapoptotic Bcl-2 protein and downregulation of the proapoptotic protein Bax, caspases 3 and 9, and p53 compared with Met-treated animals, indicating a diminution of neuronal apoptosis. These effects reflect and explain the improvement in histopathological alterations in the hippocampus of Met-treated mice. In conclusion, the beneficial effects of EGCG may be due to interconnecting pathways, including modulation of redox balance, amelioration of inflammation, and regulation of antiapoptotic proteins.

**Graphical Abstract:**

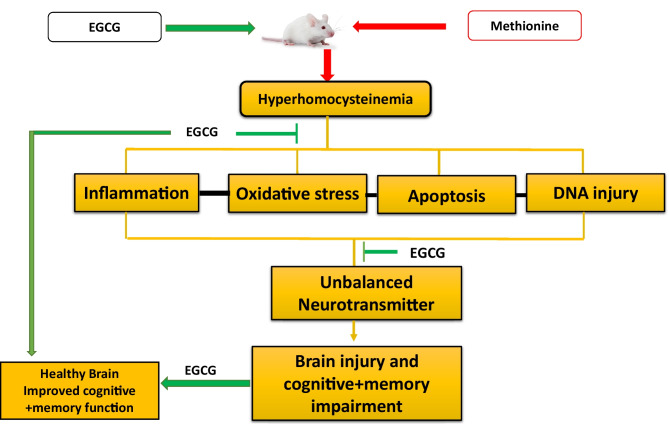

## Introduction

Cognitive impairment is a worldwide problem encountered with normal aging and early dementia. Elevated homocysteine (Hcy) is a prime risk factor in several disorders such as neurodegenerative and cardiovascular diseases) (Bhargava et al. 2018; Koklesova et al. 2021; Tawfik et al. 2021). Hyperhomocysteinemia (HHcy) is reported to be associated with cognitive dysfunction such as poor performance of spatial and verbal memory in Alzheimer’s disease) (Luzzi et al. 2021). HHcy has been reported in declining cognitive function in healthy controls and patients with mild cognitive impairment and the transition from mild cognitive impairment to dementia (Ansari et al. 2014). HHcy develops in response to excess intake of methionine (Met) (Yang et al. 2020), which is considered a reliable animal model in HHcy studies (de Rezende and D'Almeida 2014) in neurological and cardiovascular disorders (Azad et al. 2018; Zaric et al. 2019). Hcy plasma concentration is proposed as a good biomarker of dual-specificity tyrosine-phosphorylation-regulated kinase A (Dyrk1A) expression levels (Sebastiani et al. 2021). Dyrk1A is a marker of brain plasticity, and its expression can be enhanced by increased levels of amyloid-β (Aβ) (Kimura et al. 2007).

Oxidative stress plays a central role in many physiological and pathological processes. A growing body of evidence shows that the pathogenesis of HHcy-linked diseases is associated with oxidative stress (Zhang et al. 2020), inflammation (Tawfik et al. 2021; Tchantchou et al. 2021; Zhang et al. 2020), impairment of mitochondrial function, and apoptosis (Koklesova et al. 2021). Thus, scavenging excess intracellular reactive oxygen species (ROS) and blocking oxidative stress injury could be effective strategies to keep the brain healthy and prevent the development of neuronal injury, and intervention with antioxidants is effective for disease prevention and treatment.

Among various forms of tea, the health advantages of green tea (*Camellia sinensis* L.) and its metabolites have received the greatest attention from researchers (Pervin et al. 2019). Green tea has a high catechin content compared with other beverages. The polyphenolic component epigallocatechin-3-gallate (EGCG) accounts for the greatest proportion of the total catechins in green tea (Khan and Mukhtar 2018). EGCG has been found to have considerable free radical scavenging and antioxidant activity in previous studies (Lee et al. 2004). The presence of both a galloyl group and a B-ring coupled to a pyrogallol structure is thought to be responsible for the antioxidative effect of EGCG (He et al. 2018).

EGCG has the potential to treat a wide range of diseases, including diabetes, hypertension, and neurological illness, due to its anti-inflammatory and antioxidant capabilities (Wu et al. 2017). The blood–brain barrier has been reported to be permeable to EGCG (Unno et al. 2017). The neuroprotective effect of EGCG after ischemia/reperfusion-induced brain injury (Choi et al. 2004) and spinal cord injury was recently reported in a rat model (Ahadi et al. 2021). In recent studies, EGCG reduced age-related cognitive decline, improved long-term outcomes and working memory, and decreased hippocampus neuroinflammation in animal models; however, further studies are needed to explain these effects (Sebastiani et al. 2021). Therefore, the current study investigated the neuroprotective effects of EGCG against Met-induced HHcy and brain damage as well as cognitive and memory impairment.

## Materials and Methods

### Chemicals

L-methionine and EGCG were provided by Sigma (St. Louis, MO, USA). The remaining reagents were of high quality and analytical grade.

### Animals

The Egyptian Institute for Serological and Vaccine Production provided male albino mice (BALB C type) weighing 20 ± 5 g. The mice were allowed to adapt to regular laboratory conditions for 2 weeks. They were kept at a constant temperature of 25 °C ± 2 °C on a 12-h light/dark cycle and had unlimited access to commercial mouse chow and water. The Institutional Animal Ethics Committee of Mansoura University approved all research and animal welfare (approval number Sci-Z-M-2021–27).

### Experimental Design and Animal Treatment

Following acclimatization, the mice were randomly separated into four groups of six mice each. The control group did not receive any treatment. Mice in the EGCG group were given EGCG 5 mg/kg body weight orally daily for 5 weeks via gastric tube (AkdaĞ et al. 2020; El-Missiry et al. 2018b). Mice in the Met groups were supplemented with 1 g/kg body weight of Met in their drinking water for 5 weeks to induce HHcy (El-Missiry et al. 2018a). Met concentration was calculated based on the total water consumption by the mice (Tapia-Rojas et al. 2015). Mice in the Met + EGCG group were supplemented with Met and orally administered EGCG daily for 5 weeks.

### Sample Collection

The mice were anesthetized with ketamine/xylazine (0.1 mL/100 g body weight intraperitoneally) after an overnight fast (Amer et al. 2021) following the experimental period. Blood was drawn from the heart into tubes containing ethylenediaminetetraacetate as an anticoagulant, then centrifuged for 15 min at 3000 rpm. The plasma was divided into clean tubes, labeled, and refrigerated at − 20 °C. The brains were dissected immediately, and each midbrain was separated and homogenized in cold Tris–HCl buffer (0.1 M, pH 7.4). The obtained supernatant was collected, labeled, and stored at − 20 °C for further investigation.

### Behavioral Cognitive and Memory Assessment

Spatial learning changes were assessed with the open field test and the Morris water maze to determine the cognitive function and memory performance (Morris 1984). The escape latency, the number of platform locations, total swimming distance, and average speed were estimated as previously described (Giridharan et al. 2022).

### Biochemical Analysis

Quantitative determination of Hcy in the plasma and Aβ and tau protein in the brain was performed by the sandwich enzyme immunoassay technique following the instructions of the (Cat#CSB-E16551m, CEA946Mu, and MBS015010) kits purchased from Cusabio (Wuhan, China), Cloud-Clone (Houston, TX, USA), and MyBioSource (San Diego, CA, USA), respectively. The levels of glutamate and gamma-aminobutyric acid (GABA) were determined in the midbrain homogenate using enzyme-linked immunoassay (ELISA) kits (Catalog # E1716Mo and NB-E20434), provided by Bioassay Technology Laboratory (Huangpu, Shanghai, China) and Novateinbio (Woburn, MA, USA), respectively. The levels of interleukin (IL)-1β, IL-6, tumor necrosis factor (TNF)-α, IL-13, and C-reactive protein (CRP) in the plasma were determined following the instructions of the ELISA kits (Catalog # ELM-IL1b-1, ELM-IL6-1, ELM-TNFa-1, ELM-IL13-1, and CSB-E07923m) purchased from RayBiotech (Norcross, GA, USA) and Cusabio (Wuhan, China) respectively. Malondialdehyde (MDA) in brain tissue homogenate was determined spectrophotometrically as described previously using MDA kit (Catalog #MD 25 29) purchased from Biodiagnostic Company (Giza, Egypt). Protein carbonyl (PC) in the brain was determined by the colorimetric method using a PC kit (Catalog #10,005,020) from Cayman Chemical (Ann Arbor, MI, USA). The activities of superoxide dismutase (SOD), catalase (CAT), glutathione reductase (GR), and glutathione peroxidase (GPx) were spectrophotometrically estimated using kits provided by Biodiagnostic Company (Catalog #SD 25 21, CA 25 17, GR 25 23, and GP 2524) respectively. The glutathione (GSH) concentrations in the brain were assayed by the colorimetric method using a GSH kit from Biodiagnostic Company (Catalog #GR 25 11). ELISA kits for the determination of Bcl-2 (Catalog # MBS7216022), Bax (Catalog # MBS763832), caspase 3 and 9 (Catalog # MBS733100, MBS451593), and p53 (Catalog # MBS721665) were obtained from My Biosource (San Diego, CA, USA). The protocol was followed according to the manufacturer’s instructions. DNA damage in the brain was determined by the single-cell gel electrophoresis (comet assay) method, as previously reported (El-Missiry et al. 2015; Singh et al. 1988). This technique assesses DNA strand breaks in cells. Quantification of the DNA strand breaks in the obtained images was performed with CASP software to directly obtain the percentage of DNA in the tail, the tail length, and the tail moment.

### Histopathological Examination of the Hippocampus

Midbrain tissues were fixed in 10% neutral formalin (pH 7.6) for histological investigation. The samples were processed for dehydration, clearing, and paraffin infiltration then cut into 6-µm sections. Hematoxylin and eosin were used to stain the sections. Images were captured with an Amscope MU1000 camera and a bright field Olympus light microscope to look for histopathological changes in the sections using light microscopy.

### Statistical analysis

All statistical analysis were performed using GraphPad prism 8.0 software. Results are presented as mean ± the standard error of the mean (SEM) and percentage of change. Statistical comparisons were made by one-way analysis of variance (ANOVA) followed by Tukey as a post hoc test.

## Results

**Fig. 1:**
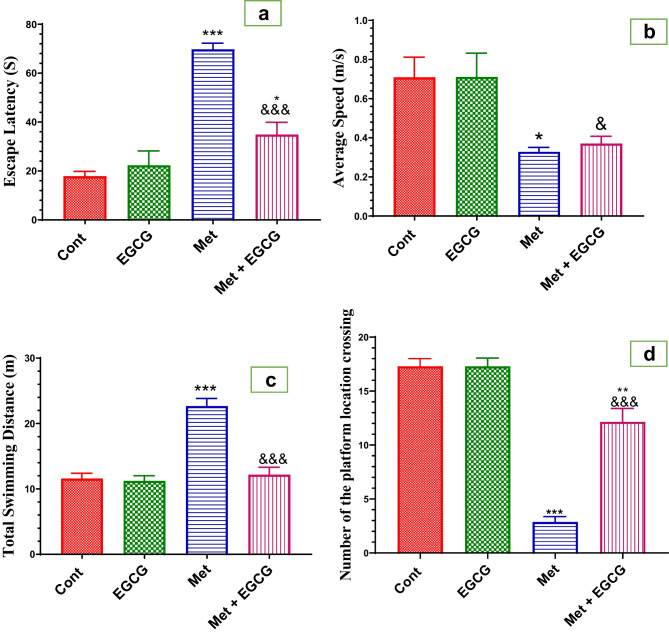
Effect Met and EGCG on escape latency (s; **a**), average speed (m/s; **b**), total swimming distance (m; **c**), and number of platform location crossings (**d**) in different mouse groups. Data are shown as mean ± SEM (*n* = 7). A single asterisk and ampersand (*, ^&^) indicates significance at *P* < 0.05, and triple asterisks and ampersands (***, ^&&&^) indicate significance at *P* < 0.001. A single asterisk and triple ampersands (*, ***) indicate comparisons with respect to the control group. A single asterisk and triple asterisks (^&^, ^&&&^) indicate comparisons with respect to the methionine group. *Cont*, control; *EGCG*, epigallocatechin-3-gallate; *Met*, methionine; *Met* + *EGCG*, methionine + epigallocatechin-3-gallate

The Morris water maze was used to test the effects of EGCG and Met on cognition and memory performance after 5 weeks of treatment (Fig. 1a–d). In comparison to the control group, daily EGCG administration had no effect on escape latency, number of platform locations, total swimming distance, or average speed. In contrast to the control group, Met treatment resulted in a significant increase in escape latency and total swimming distance, whereas significant decreases in the number of platform locations and average speed were demonstrated. EGCG coadministration effectively (*P* < 0.001) improved the cognitive and memory deficits in Met-treated mice.

**Fig. 2:**
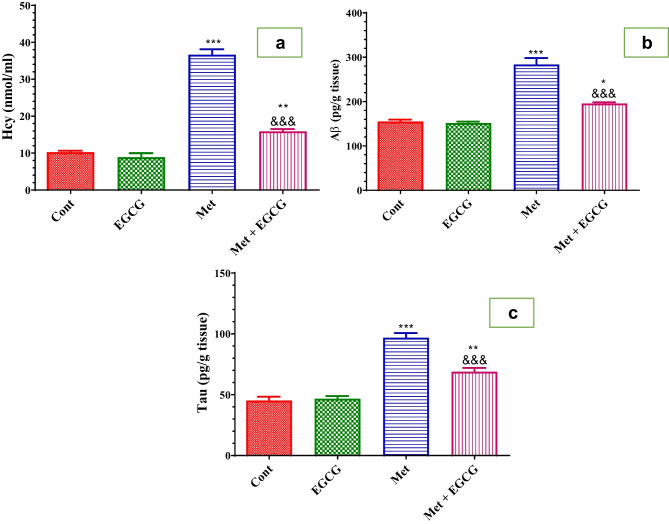
Effect of Met and EGCG on plasma levels of Hcy (nmol/mL; **a**), amyloid-β (Aβ) (pg/g tissue; **b**), and tau proteins (pg/g tissue; **c**) in different experimental groups. Data are shown as mean ± SEM (*n* = 6). A single asterisk (*) indicates significance at *P* < 0.05, double asterisk (**) indicate significance at *P* < 0.01, and triple asterisks and triple ampersands (***, ^&&&^) indicate significance at *P* < 0.001. A single asterisk, double asterisks, and triple asterisks (*, **, ***) indicate comparisons with respect to the control group. Triple ampersands (^&&&^) indicate comparisons with respect to the methionine group. *Cont*, control; *EGCG*, epigallocatechin-3-gallate; *Met*, methionine; *Met* + *EGCG*, methionine + epigallocatechin-3-gallate

Daily EGCG treatment for 5 weeks produced insignificant changes in Hcy, Aβ, and tau protein levels (Fig. 2a–c, respectively). In contrast, Met administration caused a significant increase in Hcy in the plasma and Aβ and tau protein levels in the brain (Fig. 2), whereas EGCG treatment resulted in a significant (*P* < 0.001) reduction in these levels compared with Met-treated mice (Fig. 2).

**Fig. 3:**
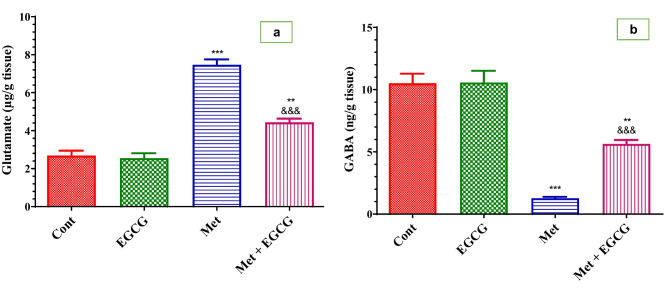
Effect of Met and EGCG on glutamate (µg/g tissue; **a**) and GABA (ng/g tissue; **b**) in the brain of all experimental groups. Data are shown as mean ± SEM (*n* = 6). Double asterisks (**) indicates significance at *P* < 0.01 and triple asterisks and triple ampersands (***, ^&&&^) indicate significance at *P* < 0.001. Double asterisks and triple asterisks (**, ***) indicate comparisons with respect to the control group. Triple ampersands (^&&&^) indicate comparisons with respect to the methionine group. *Cont*, control; *EGCG*, epigallocatechin-3-gallate; *Met*, methionine; *Met* + *EGCG*, methionine + epigallocatechin-3-gallate

Non-significant changes were observed in glutamate and GABA levels in the brain when EGCG was administered for 5 weeks (Fig. 3a and b, respectively). The Met-treated group had significant increases in glutamate and significant decline in GABA levels in the brain compared with the control group (Fig. 3a and b, respectively). EGCG supplementation inhibited the elevation of neurotransmitters in the Met + EGCG group compared with the Met-treated group.

**Fig. 4:**
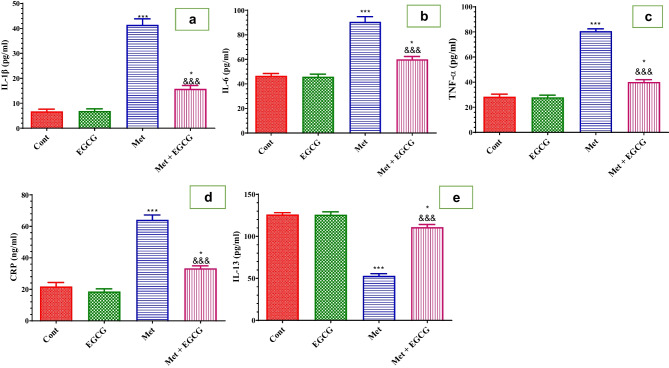
Effect of Met and EGCG on plasma levels of IL-1β (pg/ml; **a**), IL-6 (pg/ml; **b**), TNF-α (pg/ml; **c**), CRP (ng/ml; **d**), and IL-13 (pg/ml; **e**) in all experimental groups. Data are shown as mean ± SEM (*n* = 6). A single asterisk (*) indicates significance at *P* < 0.05 and triple asterisks and triple ampersands (***, ^&&&^) indicate significance at *P* < 0.001. A single asterisk and triple asterisks (*, ***) indicate comparisons with respect to the control group. Triple ampersands (^&&&^) indicate comparisons with respect to the methionine group. *Cont*, control; *EGCG*, epigallocatechin-3-gallate; *Met*, methionine; *Met* + *EGCG*, methionine + epigallocatechin-3-gallate

Figure 4 depicts the effects of Met on inflammatory and anti-inflammatory cytokines. Acute administration of Met significantly increased IL-1β, IL-6, TNF-α, and CRP levels while decreasing IL-13 levels in the plasma compared with the control. EGCG significantly (*P* < 0.001) reduced these elevated cytokine levels while increasing IL-13 levels compared with Met-treated mice. EGCG injected alone did not affect these cytokines in the plasma.

**Fig. 5:**
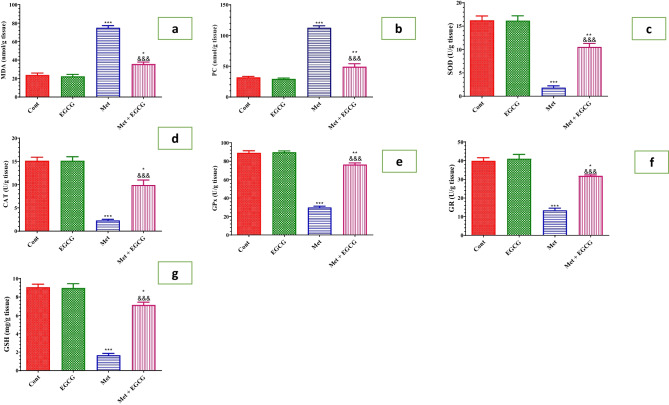
Effect of Met and EGCG on the oxidative stress markers (MDA, nmol/g tissue; **a**), protein oxidation (protein carbonyl) (PC, nmol/g tissue; **b**), and antioxidant activity of SOD (U/g tissue; **c**), CAT (U/g tissue; **d**), GPx (U/g tissue; **e**), and GR (U/g tissue; **f**), as well as GSH content (µg/g tissue; **g**) in the brains of different experimental groups. Data are shown as mean ± SEM (*n* = 6). A single asterisk (*) indicates significance at *P* < *0.05*, double asterisks (**) indicate significance at *P* < 0.01, and triple asterisks and triple ampersands (***, ^&&&^) indicate significance at *P* < 0.001*.* A single asterisk, double asterisks, and triple asterisks (*, **, ***) indicate comparisons with respect to the control group. Triple ampersands (^&&&^) indicate comparisons with respect to the methionine group. *Cont*, control; *EGCG*, epigallocatechin-3-gallate; *Met*, methionine; *Met* + *EGCG*, methionine + epigallocatechin-3-gallate

After Met-induced HHcy, oxidative stress and antioxidants were assessed in the brain (Fig. 5). Met dramatically increased MDA and PC levels (Fig. 5a and b, respectively) while lowering GSH levels as well as SOD, CAT, GR, and GPx activity (Fig. 5c–g, respectively). EGCG significantly reduced lipid peroxidation and protein oxidation and enhanced GSH content, as well as SOD, CAT, GR, and GPx activities in the brain when compared with the Met-treated group.

**Fig. 6:**
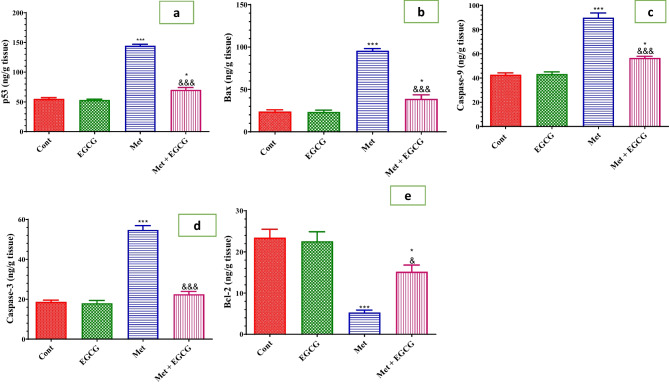
Effect of Met and EGCG on levels of p53 (ng/g tissue; **a**), Bax (ng/g tissue; **b**), caspase 9 (ng/g tissue; **c**), caspase 3 (ng/g tissue; **d**), and Bcl-2 (ng/g tissue; **e**) in the brains of all experimental groups. Data are shown as mean ± SEM (*n* = 6). A single asterisk and a single ampersand (*, ^&^) indicate significance at *P* < 0.05, and triple asterisks and triple ampersands (***, ^&&&^) indicate significance at *P* < 0.001. A single asterisk and triple asterisks (*, ***) indicate comparisons with respect to the control group. A single ampersand and triple ampersands (^&^, ^&&&^) indicate comparisons with respect to the methionine group. *Cont*, control; *EGCG*, epigallocatechin-3-gallate; *Met*, methionine; *Met* + *EGCG*, methionine + epigallocatechin-3-gallate

The regulatory proteins of the mitochondrial pathway of apoptosis were examined to further illustrate the EGCG effect on the programmed cell death mechanism in the presence of excessive Hcy and oxidative stress. In HHcy, the level of the antiapoptotic protein Bcl-2 was significantly reduced, while the levels of the proapoptotic protein p53, Bax, the initiator caspase 9, and the effector caspase 3 all increased significantly (*P* > 0.001) (Fig. 6a–e) compared with control mice. Co-administration of EGCG and Met kept these apoptotic mediator proteins at control levels. In chronic HHcy, the ability of p53 to enhance apoptosis was studied (Fig. 6a). Compared with the control group, mice with elevated Hcy had significantly higher p53 levels. The increase in p53 expression in the brain was significantly reduced (*P* > 0.001) compared with HHcy-induced mice when EGCG and Met were given together.

**Fig. 7:**
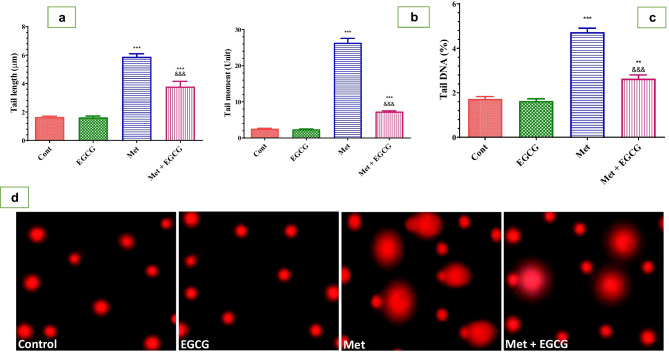
Effect of Met and EGCG on DNA damage in the brain of different experimental groups by using comet assay represented by tail length (μm; **a**), tail moment (unit; **b**), and tail DNA (%; **c**). (**d**) Representative photomicrographs of comet assay showing the effect of Met and EGCG on DNA migration in different mouse groups. Data are shown as mean ± SEM (*n* = 5). Double asterisks (**) indicate significance at *P* < 0.01 and triple asterisks and triple ampersands (***, ^&&&^) indicate significance at *P* < 0.001. Double asterisks and triple asterisks (**, ***) indicate comparisons with respect to the control group. Triple ampersands (^&&&^) indicate comparisons with respect to the methionine group. *Cont*, control; *EGCG*, epigallocatechin-3-gallate; *Met*, methionine; *Met* + *EGCG*, methionine + epigallocatechin-3-gallate

The capacity of EGCG to protect against DNA damage in mice suffering from HHcy was evaluated using the comet technique (Fig. 7). All comet parameters were considerably increased by Met, whereas EGCG protected against DNA damage and had tail DNA%, tail length, and tail moment values comparable to the control group.

**Fig. 8:**
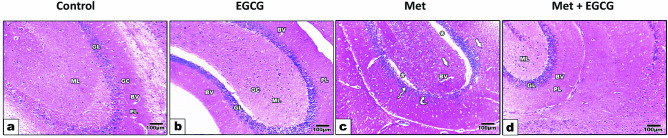
Photomicrographs of sections of the hippocampus sections of the different animal groups. **a** Sections of control mice showing normal histological appearance of hippocampus, represented by normal appearance of molecular (ML), polymorphic layers (PL), and granular layer (GL) with many glial cells (GC) among neuronal processes and blood vessels (BV); **b** hippocampal section of EGCG mice group revealing similar histological appearance to control; **c** hippocampal section of Met mice group showing disorganization and decreased thickness of pyramidal layer with cell loss (curved arrow), shrunken cells that have darkly stained nuclei and surrounded with pericellular haloes (zigzag arrows) in (PL), furthermore, separated area appears between ML and PL (asterisks). Many pyknotic cells are noticed (arrows) and dilated blood capillaries (BV) are founded as well and **d** section of Met + EGCG mice group (**d**) showing moderate to normal structure of hippocampus with increased the thickness of granular layer (GL) and almost normal cells

Histopathological assessment of the structure of the hippocampus was performed after staining with hematoxylin and eosin (Fig. 8a–d). The control and EGCG-treated mice exhibited normal cytoarchitecture of the hippocampal dentate gyrus, mice treated with Met for 5 weeks exhibited decreased thickness of cornu ammonis with shrunken of pyramidal cells layer. Moreover, dilated blood capillaries with many pyknotic cells were noted. Mice treated with Met + EGCG showed marked protection of the cornu ammonis and dentate gyrus and displayed a similar histological structure to the control group, with healthy blood capillaries inside the molecular and the polymorphic layers (Fig. 8).

## Discussion

Neurological and neurodegenerative disorders are characterized by marked disruption of neuronal structure and function. Cognitive and memory impairment can happen if disruption is not prevented. These diseases show common interconnected molecular mechanisms, including HHcy, accumulation of Aβ and tau proteins in the brain, and imbalanced neurotransmitter release. Oxidative stress, inflammation, and apoptosis in the brain are also noted. EGCG in green tea is a potent antioxidant and has shown marked beneficial effects with several diseases, including cancer (Almatroodi et al. 2020), diabetes (Othman et al. 2017; Hosseini et al. 2021), and aging (Chen et al. 2022; Fernandes et al. 2021).

However, the molecular events by which EGCG affects neuronal degeneration have not yet been fully elucidated and require further experimental studies. The present study showed that EGCG improved cognitive function and alleviated degeneration of the hippocampus by inhibiting the development of HHcy, aggregation of Aβand tau proteins, disturbance of neurotransmitters, and DNA damage in the HHcy model. This neuroprotective effect is attributed to the capacity of EGCG to inhibit the development of oxidative stress, inflammation, DNA damage, and apoptosis in the brain. These effects are confirmed by the improvement of histopathological alterations in the hippocampus and the cognitive ability of mice in the Met-induced HHcy model. EGCG has array of mechanisms indicate a remarkably broad spectrum of molecular actions performed to protect brain structure and function.

The current results showed that daily treatment with 1 g/kg body weight of Met for 5 weeks elevated Hcy levels in the blood, which agrees with a previous study showing that Hcy develops in response to excess Met intake (Yang et al. 2020). Simultaneously, concurrent supplementation with EGCG prevented HHcy development. This may be due to the ability of EGCG to maintain a regular Met cycle and increased Hcy remethylation, which could be related to the antioxidant ability of EGCG. EGCG was able to inhibit DYRK1A in the hippocampus and protect against cognitive deficits in mouse models of Down syndrome and humans by modulation of the Hcy level (De la Torre et al. 2014).

Recent study has reported that HHcy disrupts the redox balance and is involved in the development of neurodegenerative diseases and a decline in cognitive functions (Tchantchou et al. 2021). Links have been reported between elevated serum Hcy levels and behavioral and psychological signs of dementia (Kim and Lee 2014). Previous study found that elevated Hcy levels are associated with cognitive deficits (Huang et al. 2020). The decline in cognitive function may involve HHcy-induced dysfunction of the blood–brain barrier (Tawfik et al. 2021). The observations of the current study demonstrated that EGCG prevented the impairment of cognitive performance in Met-treated mice measured by the Morris water maze technique. These results agree with a recent report that EGCG and its metabolites can reduce neuronal deterioration and improve cognitive function (Pervin et al. 2019) as well as can prevent deficits of spatial memory induced by Hcy (Wang and Tian 2018). Additionally, a study found that EGCG could reduce the behavioral abnormalities associated with an animal model of Alzheimer’s disease (Walker et al. 2015). According to these studies, EGCG enhances cognitive performance and spatial memory and improves brain fitness.

The levels of Aβ and tau proteins in the brain in these animals were evaluated to explain the improvement of memory and cognitive function in mice treated with Met + EGCG. The accumulation of these proteins is associated with several neurological disorders. HHcy potentiates Aβ accumulation and neurotoxicity (Bhargava et al. 2018) by promoting the intraneural fibrillary conformation of Aβ (Baldelli et al. 2010). The present results showed that EGCG prevented the upregulation of these proteins, indicating the ability of EGCG to interfere with Aβ-peptide and tau protein metabolism by improving the Hcy level (Luo et al. 2007; Zhang et al. 2009). These findings agree with a report that EGCG significantly lowered the plasma levels of Hcy and Aβ in irradiated rats and exerted marked protection of the hippocampus (El-Missiry et al. 2018b). The lowering effect of EGCG on Aβ is compatible with other study using natural antioxidants and polyphenols (Hartman et al. 2006). The molecular mechanism of the anti-Aβ accumulation of EGCG involves production reduction, improves clearance, and inhibits Aβ fibrillogenesis (Kobayashi et al. 2020) by direct binding to natively unfolded polypeptides and promoting the formation of unstructured and nontoxic oligomers (Kobayashi et al. 2020), (Ehrnhoefer et al. 2008) and remodeling pre-formed fibrils (Fernandes et al. 2021).

These results are associated with remarkable improvement of neurotransmitter balance signified by amelioration of the glutamate and GABA levels in the brain of Met-treated rats supplemented with EGCG. EGCG rebalanced and improved the efficiency of synaptic transmission in cerebral ischemia injury by regulating excitatory and inhibitory neurotransmitter balance (Ding et al. 2012). Moreover, EGCG may act as a ligand to suppress spontaneous excitatory synaptic transmission by binding to glutamate receptors to induce anxiolytic and amnesic effects similar to those of benzodiazepine drugs (Vignes et al. 2006). An in vitro and in vivo study showed that EGCG reduced excitotoxin-induced *N*-methyl-D-aspartate production and neuronal damage in the culture system and cerebral ischemia (Lee et al. 2004). Accordingly, the data of the current study suggests that EGCG may act as a neuroprotective agent against glutamate-induced excitotoxicity in brain disorders.

The current work confirmed previous findings that HHcy is linked to oxidative stress by demonstrating that Met-induced HHcy is related to enhanced lipid peroxidation and protein oxidation as well as a considerable decrease in antioxidants (Tchantchou et al. 2021). Thus, targeting oxidative stress is suggested to be a plausible strategy to protect against brain damage and intervention with antioxidants is an effective approach. At this time, EGCG has the privilege of being an antioxidant and free radical scavenger. The present study demonstrated a significant amelioration of oxidative stress, evidenced by increased GSH content and activity of the antioxidant enzymes (SOD, CAT, GPx, and GR) in the brains of Met-treated mice after treatment with EGCG, proving its antioxidant effect. A wide range of studies has documented that EGCG has significant antioxidant properties and therapeutic potential against several disorders, including disorders of the heart, skin, and brain (Chakrawarti et al. 2016; Li et al. 2022; Zhu et al. 2014). EGCG is categorized as an antioxidant based on its chemical structure (Kim et al. 2014). In addition, EGCG can block oxidative stress by scavenging hydrogen peroxide and accordingly reducing ROS production (He et al. 2018). Targeting lipid peroxidation by EGCG may prevent alteration of the phospholipid double layer and preserve membrane stability.

Strong evidence showed that HHcy and inflammation are positively associated in several diseases, including neurodegeneration (Elsherbiny et al. 2020). Increased Hcy levels are involved in the stimulation of inflammatory mediators (e.g., adhesion molecules, leukocyte adhesion, and free radicals) (Elsherbiny et al. 2020). The present study showed a significant increase in proinflammatory cytokines (Il-1β, Il-6, CRP, TNF-α) with a remarkable decrease in the anti-inflammatory cytokine (Il-13) while treatment with EGCG prevented unbalanced cytokines. The results of the current study agree with a recent study that showed that HHcy-enhanced proinflammatory cytokines while decreasing anti-inflammatory mediators in the critical cells that form the blood–brain barrier, suggesting that it may play a role in the onset and progression of brain illnesses (Koklesova et al. 2021).

Several mechanisms have been proposed to explain the anti-inflammatory effect of EGCG. These include the following:

- Ameliorating microglia activity and hence decreasing the release of inflammatory cytokines and ROS (Dheen et al. 2007). Treatment with EGCG prevented unbalanced cytokines in Met-treated mice, indicating that EGCG may mitigate neuroinflammation by modulation of microglial activation that decreases the release of inflammatory cytokines and upregulates anti-inflammatory mediators (Cheng et al. 2021).
- EGCG was found to modulate various cell types (e.g., immune cells, vascular endothelial cells, and fibroblasts) to inhibit inflammation (Wu et al. 2017).
- EGCG likely inhibits autooxidation of Hcy because it prevents oxidative stress, leading to a decrease in ROS production and neuroinflammation (Kamat et al. 2015). These data suggest that EGCG represses inflammation likely based on its antioxidant properties.

A previous study reported that neuronal apoptosis is linked to HHcy-enhanced brain damage (Choi et al. 2004). The neuroprotective EGCG effect was further explored by assessing apoptotic-regulating proteins in the brains of HHcy-induced mice. The current study indicated that EGCG prevented the mitochondrial pathway of apoptosis, evidenced by upregulating Bcl-2 while downregulating Bax, caspase 9 and 3, and p53, indicating the antiapoptotic effect of this polyphenol. It seems that EGCG protects mitochondrial integrity against increased Hcy levels. Moreover, p53 is normalized and intact DNA becomes inactive, as demonstrated by the comet assay. EGCG has been shown to modulate apoptosis by reducing proapoptotic genes and regulating mitochondrial membrane permeabilization (Ahadi et al. 2021). EGCG protects the brain by regulating caspase 3 and poly (ADP-ribose) polymerase and possibly by modulating several signaling pathways in the brain, including PI3K/AKT/eNOS (Park et al. 2020; Nan et al. 2018). Recently, EGCG decreased Hcy-induced oxidative injury and apoptosis in endothelial cells by enhancing several signaling pathways (e.g., SIRT1/AMPK and Akt/eNOS) (Pai et al. 2021). Furthermore, EGCG has been shown to attenuate neuronal apoptosis and necroptosis in an ischemic rat brain model (Machin et al. 2021a, b) and inhibit acrylamide-induced apoptosis and astrogliosis in the cerebral cortex (He et al. 2017). The antiapoptotic effect of EGCG may be due to its antioxidant effect based on the ability of EGCG to attenuate lipid and protein oxidation and suppress the oxidized/reduced glutathione ratio in brain ischemia (Choi et al. 2004).

HHcy-induced mice showed significant DNA damage, confirming a recent study showing that chronic HHcy promotes DNA injury and induces oxidative stress in the rat brain (Dos Santos et al. 2019). Moreover, EGCG treatment attenuated DNA breaks in the brain of HHcy-induced rats, as shown by the low comet parameters observed. This implies that EGCG prevented DNA injury and maintained its coiled structure. The proposed mechanism underlying this effect may be due to the antioxidant effect of EGCG and its ability to reduce intracellular ROS levels, among other mechanisms. Thus, EGCG is a more efficient and faster free radical scavenger and antioxidant (Sebastiani et al. 2021) than vitamins E and C (Singh et al. 2016). The current data provide evidence that EGCG protects against DNA damage and neuronal toxicity caused by high Hcy levels.

Treatment with Met resulted in histopathological alterations in the hippocampal tissue. In the hippocampal tissue of HHcy-induced animals, vacuolation, multiple degenerative regions with darkly stained nuclei, and inflammatory cell infiltration, primarily lymphocytes, are the most prominent histopathological observations. These changes were averted by EGCG therapy, which was linked to the antioxidant, antiapoptotic, anti-inflammatory, and antihyperhomocysteinemia properties of EGCG. These findings support the normal spatial learning in Met-treated mice. The improvements in the histological structure of the hippocampus support the protective effect of EGCG against deterioration in spatial learning in Met-treated mice. The protection of histological structure in the brains of Met-treated mice by EGCG may be due to the alleviation of oxidative damage to neurons. EGCG may prevent disruption of the structure and function of subcellular membranes by blocking ROS overproduction and oxidative stress (Sebastiani et al. 2021). Thus, the favorable effects of EGCG on cytological and histological integrity may be related to its interactions with biological membranes due to its antilipid peroxidation properties.

**Fig. 9:**
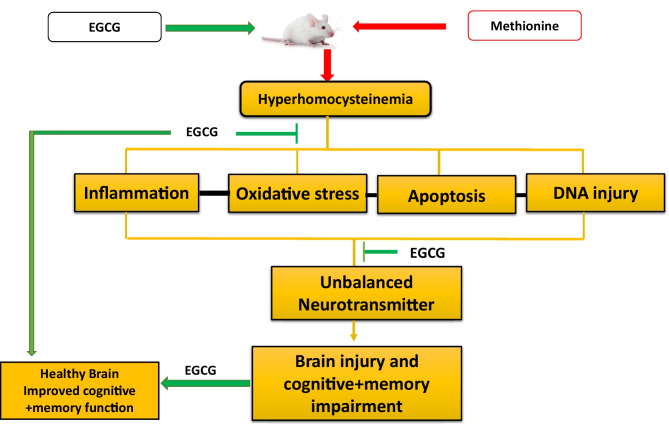
Suggested hypothesis of the suppression of cognitive function and memory impairment by EGCG in HHcy-induced brain damage. EGCG suppressed oxidative stress, inflammation DNA damage and exerted an anti-apoptotic impact by modulating mitochondrial pathway in Met-induced HHcy in rats

Taken together, it is proposed that EGCG curtails ROS induced by HHcy in stressed nervous cells resulting in alleviation of oxidative stress, inflammation, nuclear DNA damage that protect mitochondrial integrity, and downregulate apoptosis leading to the prevention of the detrimental consequences of HHcy-associated unbalanced neurotransmitters, neuro-histological changes, and improve cognitive and memory impairment (Fig. 9). The interdependent of these molecular pathways are essential for preserving brain health.

## Conclusions

In conclusion, EGCG improved cognitive and memory ability and suppressed hippocampal damage by inhibition of HHcy development, aggregation of Aβ and tau proteins, disturbance of neurotransmitter balance, and DNA defects in the HHcy model. The neuroprotective effect is due to the capability of EGCG to modulate the interdependence between oxidative stress, inflammation, apoptosis, and DNA integrity in the brain. These effects are confirmed by the improvement of histopathological alterations in the hippocampus in mice in the Met-induced HHcy model. These results confirm the therapeutic efficacy of EGCG in treating illnesses characterized by high Hcy levels.

## Data Availability

All data generated or analyzed during this study are included in this published article.
